# Impact of oat grain supplementation on growth performance, rumen microbiota, and fatty acid profiles in Hu sheep

**DOI:** 10.3389/fmicb.2025.1528298

**Published:** 2025-02-26

**Authors:** Xiaoqi Ren, Liwei Wang, Chuanzong Yu, Jianghong An, Shaoyin Fu, Hua Sun, Mengran Zhao, Rigele Te, Xiaobo Bai, Jingda Yuan, Yongbin Liu, Jiangfeng He

**Affiliations:** ^1^Research Institute of Biotechnology, Inner Mongolia Academy of Agricultural and Animal Husbandry Sciences, Hohhot, China; ^2^College of Animal Science and Technology, Inner Mongolia Minzu University, Tongliao, China; ^3^College of Life Sciences, Inner Mongolia University, Hohhot, China; ^4^College of Animal Science, Inner Mongolia Agricultural University, Hohhot, China

**Keywords:** oat grain, Hu sheep, growth performance, intestinal microbiota, SCFAs, FAs

## Abstract

The intestinal microbiota plays a vital role in animal growth and development. In this study, we explored the impact of oat grain dietary supplementation on growth performance, intestinal microbiota, short-chain fatty acids (SCFAs), and fatty acids (FAs) in Hu sheep. Thirty-two Hu lambs were randomly assigned to a control group (RC) or an oat grain-supplemented group (RO). After 90 days on their respective diets, rumen digesta were collected from six randomly selected Hu lambs per group to assess microbial diversity, SCFAs, and FAs. The RO diet significantly enhanced growth in Hu sheep (*p* < 0.01) and increased α-diversity, as indicated by Chao1 and Shannon indices. Core phyla in both groups were *Firmicutes* and *Bacteroidota*, with predominant genera including *Prevotella*, *Rikenellaceae_RC9_gut_group*, and *F082*. Oat grain supplementation led to significant shifts in microbial composition, increasing the abundance of *Acidobacteriota*, *Proteobacteria*, *Chloroflexi*, *Actinobacteriota*, and *Subgroup_2,* while decreasing *Bacteroidota* and *Oscillospiraceae* (*p* < 0.05). The RO group also exhibited lower levels of isobutyric and citraconic acids but higher levels of azelaic acid (*p* < 0.05). These results indicate that oat grain supplementation enhances beneficial rumen microbes and optimizes FAs and SCFAs composition, thereby promoting weight gain in Hu sheep.

## 1 Introduction

The rising demand for mutton has spurred research focused on enhancing the growth performance, meat quality, and nutritional value of mutton sheep. In recent years, China’s livestock industry has shifted from traditional free grazing to large-scale, intensive breeding. This shift has significantly improved production efficiency and product quality while reducing costs and environmental impact. Concentrate feeds are a pivotal nutrient source in intensive mutton breeding, relying on high-calorie grains like corn. However, corn protein has an imbalanced amino acid profile, particularly low in lysine (Liu et al., 2020). Lysine, the first-limiting amino acid for sheep, is crucial for development and immune function. This deficiency hampers protein synthesis, fails to meet essential amino acid needs, and must be supplemented by other dietary components, reducing feed conversion and affecting yield and meat quality. Oat grain, with a superior amino acid profile and higher lysine content than other cereals (Nieto-Nieto et al., 2014), has shown high yield, palatability, and nutrient richness as livestock feed (Andrzejewska et al., 2019). It positively affects ruminant performance, meat quality (Salgado et al., 2013; Schulmeister et al., 2020), and gastrointestinal health (An et al., 2020). Due to its high nutritional value, low cost, and ease of storage, oat grain has gained attention as a feedstuff. Rich in protein, fiber, and nutrients, especially β-glucan with immunomodulatory functions, oat can reduce colonic inflammation, enhance barrier function, and elevate intestinal SCFAs levels (Cheng et al., 2021). Oat also exhibits probiotic properties, stimulating beneficial gut bacteria growth, particularly *Lactobacillus*, and *Bacteroidetes*, increasing SCFAs (Metzler-Zebeli et al., 2011; Murphy et al., 2012), supporting healthy gut microbiota (Tosh and Bordenave, 2020). Furthermore, oats contain bioactive compounds like polyphenols and antioxidants, benefiting gut health. Flavonoids in oats can modulate microbial population, increasing *Akkermansia*, which has antihyperlipidaemic effects (Duan et al., 2021). Oats improve lipid metabolism and balance gut microflora, significantly boosting SCFAs, including butyric acid, in the colon (Wang et al., 2022). Dietary fiber and phytonutrients in oats can increase milk production in dairy cows while reducing CH_4_ emissions (Ramin et al., 2021).

Currently, the impact of oat grain on sheep gut microbiota, particularly in regulating microbiota diversity and function, remains poorly understood. Oat grain processing generates significant residues, often seen as having limited economic value. In this experiment, we used this portion of unsalable oat grain. These oat grains include small and broken grains, which are usually unsalable in the market due to their uneven texture. Although these parts may not meet consumer preferences in appearance, they still have good quality in terms of nutritional content. To optimize resource utilization, minimize waste, and enhance agricultural sustainability, unsalable oat grains were used to substitute for corn in concentrate feed. This study aimed to investigate the effects of an oat grain-based diet on growth performance, intestinal microbiota, FAs, and SCFAs in Hu sheep, focusing on microbiota composition changes. Understanding how oat grains influence Hu sheep gut microbiota can provide insights into dietary strategies promoting gut health and productivity in sheep farming.

## 2 Materials and methods

### 2.1 Animals, diets, and experimental design

Thirty-two Hu sheep with similar nutritional status (average body weight of 27.28 ± 0.83 kg, approximately 120 days old) were selected and divided into two groups. The control group (RC) received corn grain as the primary concentrate feed. The experimental group (RO) was fed a mixed concentrate diet with oat grain, maintaining a ratio of 35:65 (DM basis). In the RO group, unsalable oat grain partially replaced maize, resulting in an oat grain content of 35% (DM basis). The nutrient composition of the diets is detailed in Table 1. Throughout the experiment, sheep had ad libitum to roughage and water. And they were raised under the same environmental conditions. During the experiment, both groups of sheep were kept under the same temperature and humidity conditions to ensure consistency in the experimental conditions. The study spanned 100 days, comprising a 10-day adaptation period followed by a 90-day experimental phase. After the end of the experiment, six Hu lambs from each group were randomly selected for slaughter. The experimental protocol was approved by the professional committee of the Academy of Agricultural and Animal Husbandry Sciences of Inner Mongolia Autonomous Region and the Animal Welfare Association (No. IMAAAHS-2023-21).

**Table 1:** The dietary nutrition levels of RC and RO groups.

Dietary composition (%)	RC	RO
Oat grain	0	35
Corn	70	35
Soybean meal	24	24
Ca(HCO_3_)_2_	1	1
NaCl	1	1
NaHCO_3_	1	1
Premix^1^	3	3
Nutrient levels (%)		
Dry matter (DM)	93.8	93.92
Ash	7.65	4.15
Crude protein	18.57	18.22
Crude fat	1.32	3.85
Calcium	0.43	0.16
Phosphorus	0.54	0.5
Neutral detergent fiber	16.05	13.77
Acid detergent fiber	4.04	2.63
Acid detergent lignin	0.38	0.71
Water-soluble carbohydrate	35.68	26.35
Energy	16.04 KJ/g	16.55 KJ/g

1 In basic diets, Premix offered the following per kilogram: vitamin A (280,000 IU), vitamin D (350,000 IU), vitamin E (2,500 IU), D-biotin (50 mg), β-carotene (25 mg), Fe (750 mg), Cu (250 mg), Mn (1,400 mg), Zn (3,500 mg), Co (30 mg), I (55 mg), Se (30 mg), and ethoxyquin (500 mg).

### 2.2 Growth performance measurement

Each lamb was weighed every 30 days before morning feeding to calculate the average daily gain (ADG). Throughout the experiment, both the feed offered and any surplus feed were precisely weighed and recorded daily to estimate the average daily feed intake (ADFI).

### 2.3 Sample collection

Prior to slaughter, veterinary examinations confirmed the lamb’s good health. Post-slaughter, rumen contents were collected consistently from all lambs, ensuring uniform sampling locations. To minimize contamination, samples were handled aseptically whenever possible. Rumen contents were placed in 50 ml aseptic, enzyme-free centrifuge tubes for subsequent analysis of the gut microbiota, SCFAs, and FAs composition. Samples from both the RC group (RC1-RC6) and the RO group (RO1-RO6) were immediately frozen in liquid nitrogen and stored at −80°C.

### 2.4 Extraction of microbiota DNA

The hexadecyl trimethyl ammonium bromide (CTAB) method was used to extract total microbial DNA from the samples. The concentration and purity of the extracted DNA were assessed using electrophoresis on 1% agarose gels. The DNA was then diluted to a concentration of 1 ng/μl with sterile water.

### 2.5 Amplicon generation and 16S rRNA gene sequencing

Specific regions of the 16S rRNA genes, including 16S V4, 16S V3, 16S V3-V4, and 16S V4-V5, were amplified using designated primers (16S V4, 515F-806R) with barcodes. Each PCR reaction included 15 μl of Phusion® High-Fidelity PCR Master Mix (New England Biolabs), 0.2 μM of each primer, and 10 ng of target DNA. The PCR involved 30 cycles of denaturation at 98°C for 10 s, annealing at 50°C for 30 s, and extension at 72°C for 30 s, followed by a final extension at 72°C for 5 min. For DNA detection, an equal volume of 1 × loading buffer (containing SYB green) was combined with the PCR products and subjected to electrophoresis on a 2% agarose gel. The PCR products were then mixed in equal proportions and purified using a Qiagen Gel Extraction Kit (Qiagen, Germany). Libraries for sequencing were constructed with NEBNext® Ultra™ IIDNA Library Prep Kit (Cat No. E7645). Library quality was assessed using the Qubit@2.0 Fluorometer (Thermo Scientific) and Agilent Bioanalyzer 2100 system. Sequencing was performed on the Illumina NovaSeq platform, generating 250 bp paired-end reads.

### 2.6 Bioinformatics and statistical analysis

Paired-end reads were assigned to samples based on their unique barcodes and truncated to remove the barcodes and primer sequences. Reads were merged using FLASH (Version 1.2.11), and splicing sequences were termed Raw Tags. Fastp (Version 0.20.0) ensured high-quality data and clean tags were aligned against the Silva database via Vsearch (Version 2.15.0) to identify and remove chimeric sequences, yielding effective tags. We conducted α-diversity analysis on normalized amplicon sequence variants (ASVs) using five indices (observed ASVs, Chao1, Simpson, Shannon, and Good’s coverage) in QIIME2 to evaluate species diversity complexity. Also, β-diversity analysis involved calculating unweighted unifrac distances in QIIME2. Principal coordinate analysis (PCoA) was performed using the ade4 and ggplot2 packages in R (version 2.15.3) to reduce dimensionality. A T-test analysis in Version 3.5.3 identifies species with significant differences at each taxonomic level (Phylum, Class, Order, Family, Genus). LEfSe analysis, using LEfSe software (Version 1.0) with an LDA score threshold of 4, identified potential biomarkers. Tax4Fun software was used for function annotation analysis. Spearman correlation analysis in R (Version 2.15.3) was conducted using the psych and pheatmap packages. SPSS software (IBM SPSS 26.0) analyzed growth performance and FAs data, with significance reported at *p* < 0.05. T-tests assessed significant differences.

### 2.7 Determination of fatty acids

Rumen content samples were thawed on ice to minimize degradation. A 20 μl of rumen content samples were added to a 96-well plate and transferred to the Eppendorf epMotion Workstation. Then, 120 μl of ice-cold methanol containing internal standards was automatically added to each sample and vortexed for 5 min. The plate was centrifuged at 4,000 *g* for 30 min. After centrifugation, 30 μl of supernatant was transferred to a clean 96-well plate, and 20 μl of freshly prepared derivatization reagent was added to each well. Derivatization was performed at 30°C for 60 min. Following this, 330 μl of ice-cold 50% methanol solution was added to dilute the samples. The plate was stored at −20°C for 20 min and then centrifuged at 4,000 *g* for 30 min at 4°C. Finally, 135 μl of supernatant was transferred to a new 96-well plate, ensuring each well contained 10 μl of internal standard. Serial dilutions of derivatized stock standards were added to the left wells. The plate was sealed for LC–MS analysis.

An ultra-performance liquid chromatography coupled to tandem mass spectrometry (UPLC-MS/MS) system (ACQUITY UPLC-Xevo TQ-S, Waters Corp., Milford, MA, USA) was utilized to quantitate all targeted metabolites in Novogene Co., Ltd. (Beijing, China). Samples were injected onto an ACQUITY UPLC BEH C18 1.7 μM VanGuard pre-column (2.1 × 5 mm) and an ACQUITY UPLC BEH C18 1.7 μM analytical column (2.1 × 100 mm) using an 18-min linear gradient at a flow rate of 0.4 ml/min for the positive/negative polarity mode. The mobile phases consisted of eluent A (0.1% Formic acid in water) and eluent B (acetonitrile: IPA = 70:30). The gradient program was as follows: 0–1 min (5% B), 1-11 min (5–78% B), 11–13.5 min (78–95% B), 13.5–14 min (95–100% B), 14–16 min (100% B), 16–16.1 min (100–5% B), and 16.1–18 min (5% B). The Xevo TQ-S mass spectrometer was operated in positive (negative) polarity mode with the following settings: capillary voltage of 1.5 (2.0) KV, source temperature of 150°C, desolvation temperature of 550°C, and desolvation gas flow of 1,000 L/Hr.

The detection of the experimental samples via MRM (Multiple Reaction Monitoring) was based on the novogene self-built method. The Q1, Q3, RT (retention time), DP (declustering potential), and CE (collision energy) were used for the metabolite identification. The ratio of the Q3 peak area of the compound to that of the internal standard was brought into the standard curve. The concentration of the compound was calculated from the known internal standard concentration. The data files generated by UPLC-MS/MS were processed using the MassLynx Version 4.1 for peak integration and correction.

### 2.8 Determination of short-chain fatty acids

The rumen content samples were thawed on ice, and 200 μl was pipetted into 2 ml glass centrifuge tubes. Subsequently, 900 μl of 0.5% phosphoric acid was added, and the mixture was shaken and vortexed for 2 min. The tubes were centrifuged at 14,000 *g* for 10 min. Afterward, 800 μl of the supernatant was transferred to a new tube, and an equal volume of ethyl acetate was added. This mixture was again shaken and centrifuged at 14,000 *g* for 10 min. Then, 600 μl of the resulting supernatant was collected, and 4-methylpentanoic acid was added to achieve a final concentration of 500 μM as an internal standard. The solution was mixed and transferred to a sample vial for Gas chromatography–mass spectrometry (GC–MS) analysis. The injection volume was set at 1 μl with a split ratio of 10:1. Standard substances of acetic acid, propionic acid, butyric acid, isobutyric acid, valeric acid, isovaleric acid, and hexanoic acid were prepared with concentration gradients ranging from 0.1 to 100 μg/ml in ethyl acetate. Specifically, the concentrations were set at 0.1, 0.5, 1, 5, 10, 20, 50, and 100 μg/ml. A volume of 600 μl of each standard solution was combined with 25 μl of 4-methylpentanoic acid as the internal standard. The mixture was then transferred into a sample vial for subsequent analysis by GC–MS.

The samples were separated on an Agilent DB-WAX capillary column (30 m × 0.25 mm ID × 0.25 μm) within a GC system. The heating process started at 90°C and increased to 120°C at a rate of 10°C/min. Subsequently, the temperature rose to 150°C at 5°C/min. Finally, it escalated to 250°C at 25°C/min and remained for 2 min. The carrier gas (helium) flow rate was 1.0 ml/min. Quality control (QC) samples were interspersed within the sample queue at regular intervals to ensure system stability and repeatability. Mass spectrometry analysis was performed using the Agilent 7890A/5975C GC–MS system.

## 3 Results

### 3.1 Growth performance

**Figure 1:**
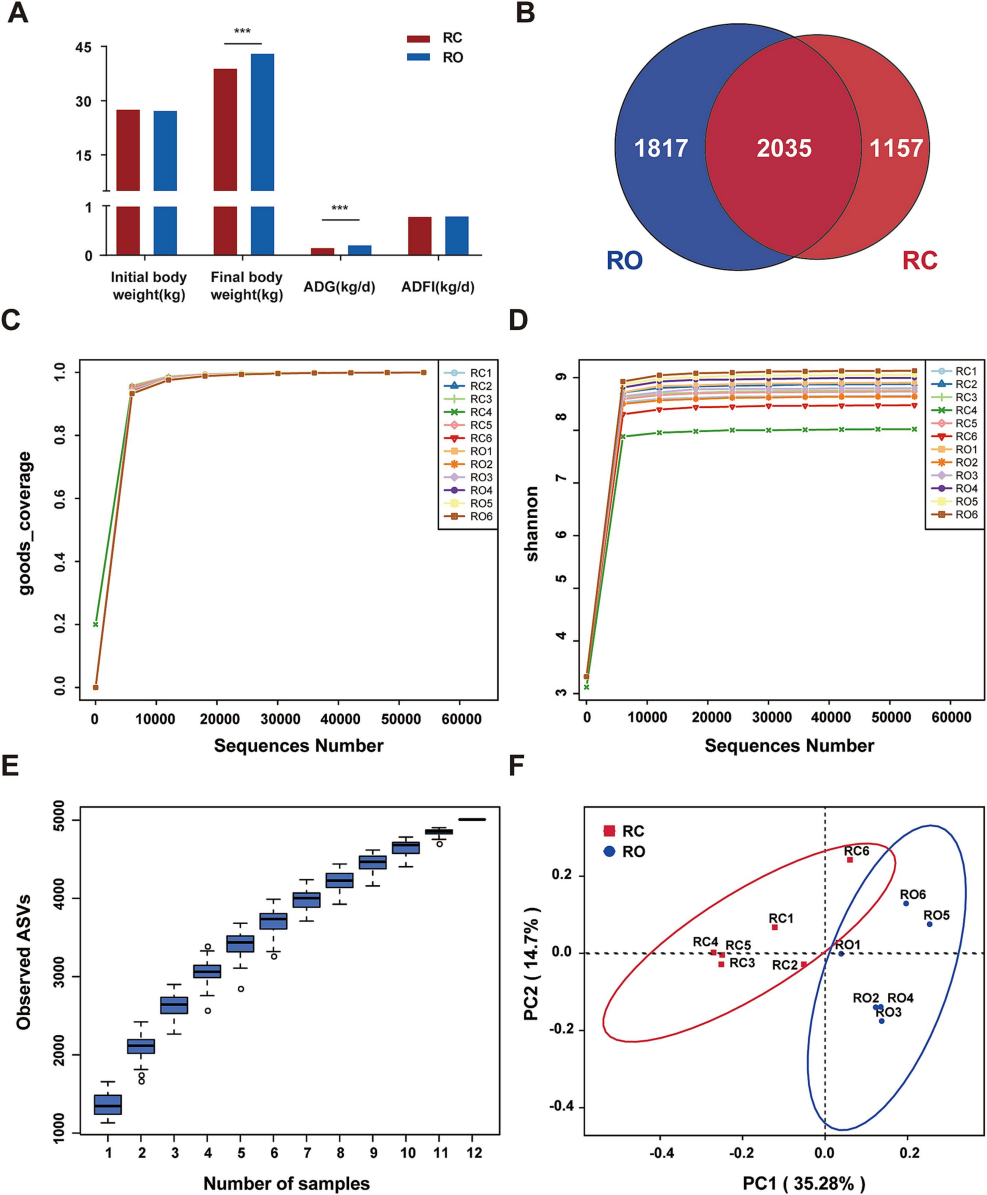
**(A)** Effects of oat grain diet supplementation on growth performance of Hu sheep. DNA sequence data analysis, **(B)** Venn diagram, the numbers in the figure show the unique or shared ASVs of each group; **(C)** It shows the sequencing depth of the RC and RO groups; **(D)** Rarefaction of the different samples; **(E)** The species accumulation curves of each group of samples; **(F)** Differences in Principal Coordinate Analysis (PCoA) of intestinal microbiome RC and RO groups. The red dots represent the samples of the RC group and the blue dots represent the samples of the RO group. The distance between the two points represents the difference in the intestinal microbiota.

The initial body weights of Hu sheep were not significantly different between the RC and RO groups. However, the RO group exhibited higher final body weights compared to the RC group. The average final weight of Hu sheep in the RC group was 38.83 kg, whereas that in the RO group was 42.97 kg, making a 10.66% increase over the control group (*p* < 0.001). Additionally, the average daily gain (ADG) was greater in the RO group (0.2 kg/d) compared to the RC group (0.14 kg/d; *p* < 0.001). Although the average daily feed intake (ADFI) of Hu sheep in the RO group tended to be higher than in the RC group, this difference was not statistically significant (*p* > 0.05; Figure 1A).

### 3.2 DNA sequences analysis

In this study, 16S rRNA gene sequencing was conducted on 12 Hu sheep samples, generating a total of 964,519 sequences. After chimera checks and filtering, we obtained 776,482 high-quality sequences across all samples, averaging 64,706 sequences per sample. Additional details are provided in Supplementary Table S1. Taxonomic assignment revealed 5,009 ASVs at 100% nucleotide sequence similarity. Specifically, the RC and RO groups produced 3,192 and 3,852 ASVs, respectively (Figure 1B). Notably, all samples in both groups exhibited Good’s Coverage index values of 1 (Figure 1C). The rarefaction curves stabilized when the number of effective sequences exceeded 5,000, indicating adequate sequencing depth and quantity (Figure 1D). Furthermore, the species accumulation curves for each sample group displayed a relatively flat trend at the extremity, suggesting an adequate sample size (Figure 1E). These findings collectively validate the comprehensiveness and representativeness of our sequencing data.

### 3.3 Microbial diversity index analysis

To evaluate the α-diversity of the microbial community, we used the Chao1, Shannon, and Simpson indices to assess species richness and community diversity. The average Chao1 and Shannon indices for the RC group were 1,257.09 and 8.59, respectively, while the RO group showed significantly higher values at 1476.63 and 8.92 (*p* < 0.05, Table 2). Specifically, both indices were markedly higher in the RO group compared to the RC group. Although the Simpson index averaged 0.991 in the RC group and 0.994 in the RO group, this difference was not statistically significant. Additional details are provided in Supplementary Table S2. The PCoA (Figure 1F), based on unweighted Unifrac distances, reflected the species composition and relative abundances of ASVs in the samples. A clear separation between the RC and RO groups was observed, suggesting that incorporating oat grain into the diet led to a significant divergence in the structure of the gut microbiota.

**Table 2:** The alpha diversity index between the RC and RO groups.

Name	RC	RO	*p*
Chao1	1257.09	1476.63	0.03
Shannon	8.59	8.92	0.04
Simpson	0.991	0.994	0.21

### 3.4 Bacterial community composition at different taxonomical levels

**Figure 2:**
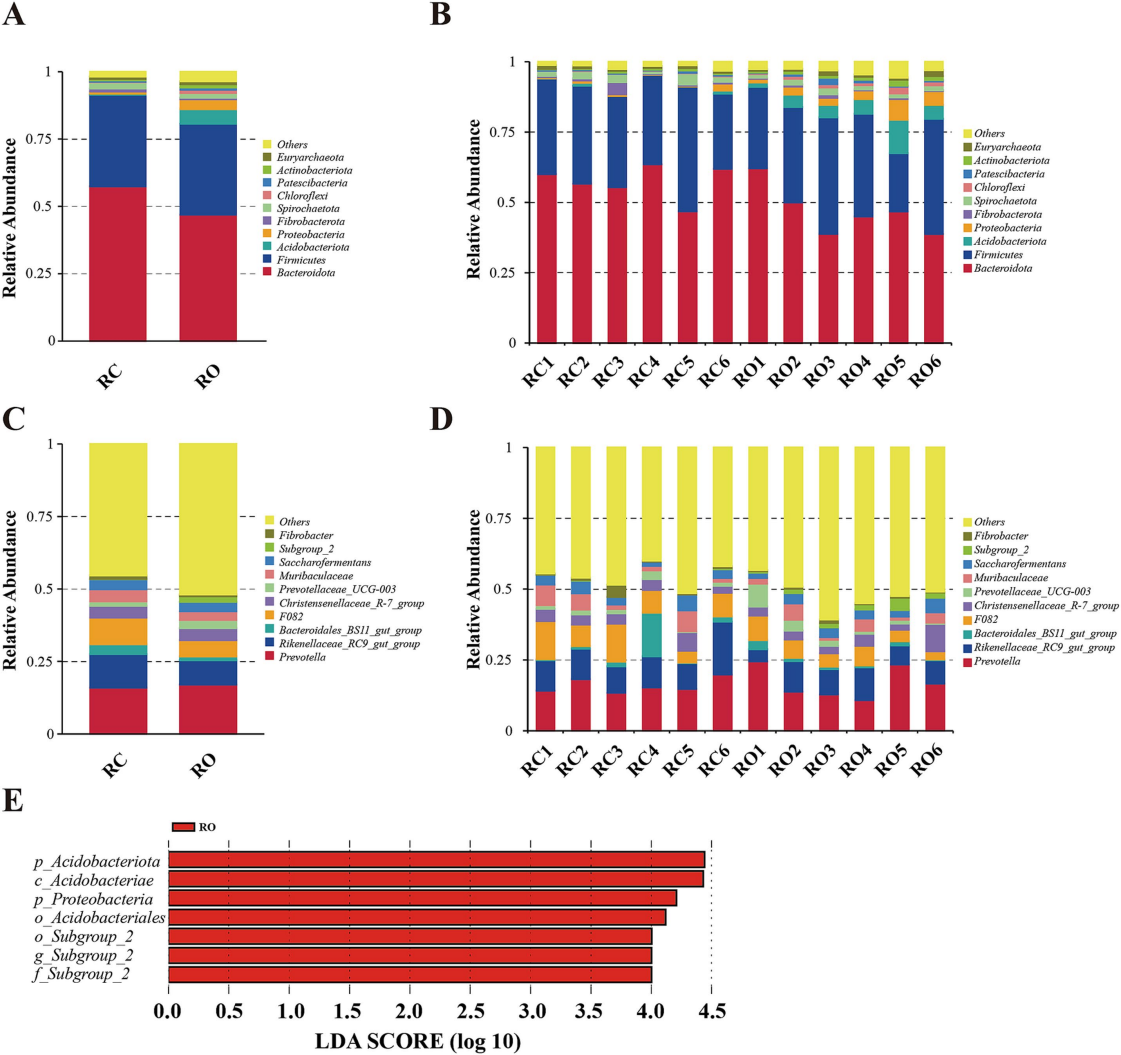
The taxonomic distribution between RC group and RO group samples (each color represents the relative abundance of a taxonomic bacterium). **(A)** at phylum level (top 10). **(B)** Between-group at the phylum level (top 10). **(C)** at genus level (top 10). **(D)** Between-group at the genus level (top 10). **(E)** A significant differentiation of bacterial tax between RC and RO groups was determined by LEfSe. LDA scores were calculated for bacterial tax that were differentially enriched between different groups.

We quantified the relative proportions of predominant taxa at both the phylum and genus levels based on the distribution of microbial taxa in different groups. As shown in Figure 2, the top 10 phyla were *Bacteroidota*, *Firmicutes*, *Acidobacteriota*, *Proteobacteria*, *Fibrobacterota*, *Siprochaetota*, *Chloroflexi*, *Patescibacteria*, *Actinobacteriota*, and *Euryarchaeota*. Notably, *Bacteroidota* were the most abundant phylum across all samples, with *Firmicutes* being the second most abundant. In the RC group, *Bacteroidota* and *Firmicutes* accounted for 91.43% of the relative abundance, with *Bacteroidota* at 57.40% and *Firmicutes* at 34.03%. Similarly, these two phyla retained the highest relative abundance in the RO group, comprising 46.90% (*Bacteroidota*) and 33.76% (*Firmicutes*; Supplementary Table S3). Compared to the RC group, the RO group exhibited a significant increase in the abundance of *Acidobacteriota*, *Proteobacteria*, *Chloroflexi*, and *Actinobacteriota* (*p* < 0.05). The abundance of *Patescibacteria* increased, but the difference was not statistically significant (*p* = 0.287). In contrast, the abundance of *Bacteroidota* significantly decreased (*p* < 0.05), while the abundance of *Siprochaetota* also decreased, though not significantly (*p* = 0.25).

At the genus level, the top 10 genera were *Prevotella*, *Rikenellaceae_RC9_gut_group*, *Bacteroidales_BS11_gut_group*, *F082*, *Christensenellaceae_R-7_group*, *Prevotellaceae_UCG-003*, *Muribaculaceae*, *Saccharofermentans*, *Subgroup_2*, and *Fibrobacter*. The core genera of the RC group included *Prevotella* (15.94%), *Rikenellaceae_RC9_gut_group* (11.61%), *F082* (9.16%), *Muribaculaceae* (4.13%), *Christensenellaceae_R-7_group* (4.07%), *Saccharofermentans* (3.48%), and *Bacteroidales_BS11_gut_group* (3.41%). In the RO group, the genera with high relative abundance were *Prevotella* (16.99%), *Rikenellaceae_RC9_gut_group* (8.42%), *F082* (5.61%), *Christensenellaceae_R7_group* (4.18%), and *Saccharofermentans* (3.26%; Supplementary Table S4). Compared to the RC group, the RO group exhibited a significant increase in the abundance of *Subgroup_2* (*p* < 0.05). Although the abundances of *Prevotella*, *Bacteroidales_BS11_gut_group*, *Christensenellaceae_R-7_group*, and *Prevotellaceae_UCG-003* also increased, these differences were not statistically significant. On the other hand, the abundance of *Rikenelaceae_RC9_gut_group*, *F082*, *Muribaculaceae*, *Saccharofermentans*, and *Fibrobacter* was reduced in the RO group, although the difference was not significant.

Differences between the RC and RO groups were examined using LEfSe analysis (LDA > 2) across all taxonomic levels. Seven significant differences were identified, with the RO group showing enrichment in two phyla (*Acidobacteriota* and *Proteobacteria*), one class (*Acidobacteriae*), two orders (*Acidobacteriales* and *Subgroup_2*), one family (*Subgroup_2*), and one genus (*Subgroup_2*).

### 3.5 Microbial function prediction

**Figure 3:**
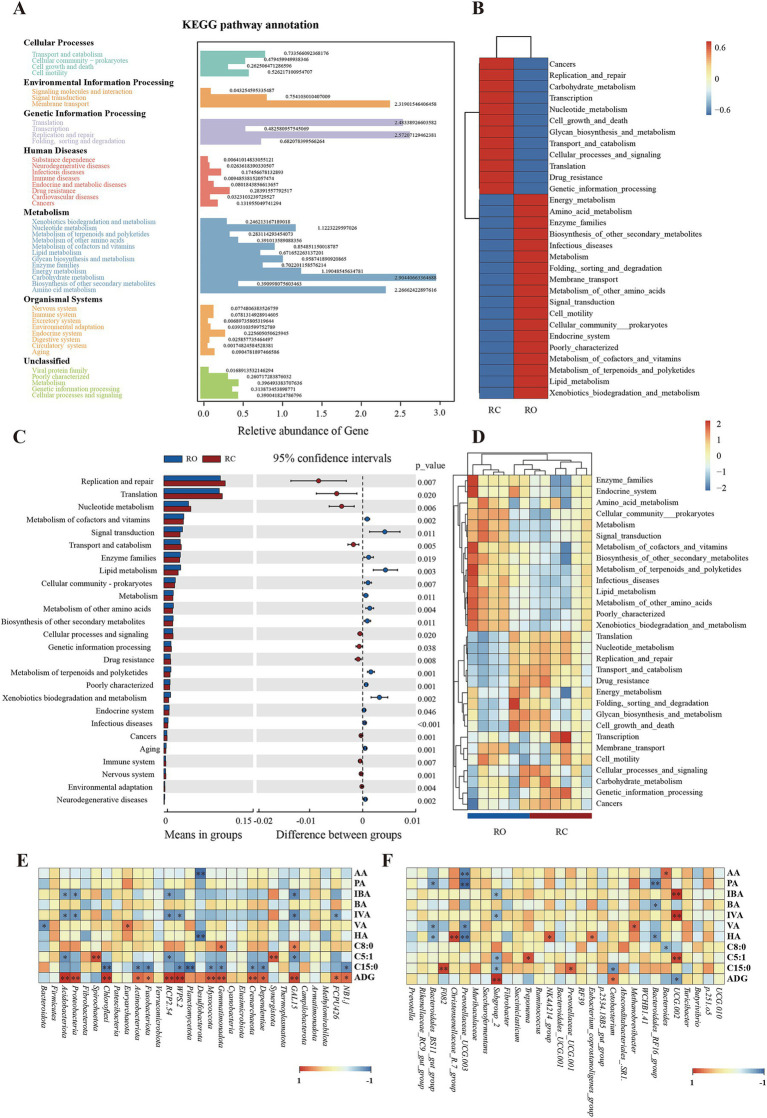
Genomic functional predictions. **(A)** KEGG pathway annotation. **(B)** Heatmap clustered based on functional predictions of the different groups in the level 2 pathway. **(C)** Comparison of RC and RO groups in differential metabolic pathways. **(D)** Heatmap clustered based on functional predictions of each sample in the level 2 KEGG pathway. The results of Spearman’s analysis show SCFAs FAs and ADG in the longitudinal direction and each bacterial information in the transverse direction. **(E)** at the phylum level. **(F)** at the genus level. The value corresponding to the intermediate heatmap is the Spearman correlation coefficient r, which is between −1 and 1, r < 0 is a negative correlation, r > 0 is a positive correlation, and marked * indicates significance test *p* < 0.05.

In this study, Tax4fun was used to predict and investigate the molecular functions of microbial communities in two sample sets. As shown in Figure 3A, 44 functional genes are associated with various biological pathways, including cellular processes, environmental processing, genetic processing, human diseases, metabolism, and organismal systems pathways. At the Kyoto Encyclopedia of Genes and Genomes (KEGG) level 2, we identified 44 functional genes across the RC and RO groups. These genes predominantly pertained to carbohydrate metabolism, replication and repair, translation, membrane transport, amino acid metabolism, and energy metabolism. Further analysis revealed that the gut microbiota in the RO group mainly focused on metabolic pathways such as cofactor and vitamin metabolism, terpenoid and polyketide metabolism, other amino acid metabolism, the endocrine system, lipid metabolism, and enzyme families. In contrast, intestinal microbes in the RC group primarily focused on nucleotide metabolism, replication and repair, transport and catabolism, drug resistance, and translation (Figures 3B,D).

We identified significant changes in KEGG pathways in the gut microbiota between the two groups using the T-test. Specifically, the RO group exhibited a higher abundance of genes related to cofactor and vitamin metabolism, lipid metabolism, amino acid metabolism, terpenoids, and polyketide metabolism compared to the RC group (*p* < 0.05). However, the RO group had significantly fewer genes involved in replication and repair, cellular processes, and signaling (*p* < 0.05; Figure 3C).

### 3.6 Fatty acids composition

Table 3 details the impact of oat grain on the fatty acids composition in the rumen of Hu lambs. A total of 25 FAs were identified, including 24 saturated fatty acids (SFAs), 10 monounsaturated fatty acids (MUFAs), and one polyunsaturated fatty acids (PUFAs). The predominant FAs were azelaic acid (C9:0), oleic acid (C18:1), and caprylic acid (C8:0). The RO group showed lower levels of SFAs and MUFAs compared to the RC group, while the PUFAs content was higher, though the difference was not statistically significant. Among SFAs, azelaic acid (C9:0) was significantly different between the two groups (*p* < 0.05), with concentrations of 38.94 μmol/L in the RC group and 54.28 μmol/L in the RO group. Indicating a significantly higher level in the RO group. For MUFAs, citraconic acid (C5:1) showed a significant difference (*p* < 0.05), with concentrations of 0.02 μmol/L in the RC group and 0.01 μmol/L in the RO group, indicating a significantly lower level in the RO group. Additionally, pentadecanoic acid (C15:0) was significantly lower in the RO group compared to the RC group. Further details are provided in Supplementary Table S5.

**Table 3:** Effects of oat grain diet supplementation on fatty acid contents in the rumen of Hu sheep.

Name	RC (μmol/L)	RO (μmol/L)	*p*
Azelaic acid	38.94	54.28	0.02
Citraconic acid	0.02	0.01	0.05
Pentadecanoic acid	3.30	2.46	0.06
Tridecanoic acid	0.69	0.52	0.15
Myristic acid	3.53	2.79	0.17
Decanoic acid	0.79	0.61	0.20
Heptanoic acid	9.16	7.08	0.21
Dodecanoic acid	1.74	1.15	0.22
Itaconic acid	0.24	0.43	0.28
2,2-Dimethyladipic acid	0.42	0.38	0.42
alpha-Linolenic acid	2.07	1.76	0.43
10-Trans-Heptadecenoic acid	0.16	0.14	0.44
Octanoic acid	13.08	12.30	0.45
Undecanoic acid	0.17	0.15	0.46
12-Tridecenoic acid	0.01	0.01	0.51
Methylsuccinic acid	3.87	4.20	0.51
3-Methyladipic acid	2.85	3.43	0.56
Nonanoic acid	1.41	1.51	0.56
Adipic acid	7.25	8.42	0.57
Oleic acid	19.72	15.43	0.61
Pimelic acid	2.70	2.97	0.65
5Z-Dodecenoic acid	0.11	0.09	0.67
Citramalic acid	0.34	0.32	0.68
Sebacic acid	8.14	8.89	0.73
9E-tetradecenoic acid	0.06	0.06	0.73
12-Hydroxystearic acid	0.77	0.74	0.79
2-Butenoic acid	3.88	4.27	0.83
4-Methylhexanoic acid	2.13	1.32	0.87
2-Methylhexanoic acid	0.02	0.02	0.88
Suberic acid	5.31	5.47	0.88
Palmitoleic acid	1.06	0.93	0.91
Methylglutaric acid	0.89	0.88	0.93
2,2-Dimethylsuccinic acid	0.90	0.90	0.94
10Z-Nonadecenoic acid	0.10	0.08	0.98
2-Hydroxycaproic acid	3.06	2.98	1.00
SFAs	111.53	123.89	0.85
MUFAs	25.31	21.35	0.84
PUFAs n-3	2.07	1.76	0.58

### 3.7 Short-chain fatty acids composition

The concentration of isobutyric acid was significantly lower in the RO group (35.88 μg/ml) compared to the RC group (47.80 μg/ml; *p* < 0.05). In contrast, the levels of butyric acid and valeric acid were higher in the RO group, although the differences were not statistically significant (*p* > 0.05). Additionally, the levels of acetic acid, propionic acid, isovaleric acid, and hexanoic acid were lower in the RO group compared to the RC group, but these differences were not significant (Table 4). Further details are provided in Supplementary Table S5.

**Table 4:** Effects of oat grain diet supplementation on SCFAs contents in the rumen of Hu sheep.

Name	RC (μg/ml)	RO (μg/ml)	*p*
Acetic acid	434.20	361.68	0.10
Propionic acid	260.64	247.22	0.37
Isobutyric acid	48.33	35.88	0.01
Butyric acid	186.72	207.66	0.26
Isovaleric acid	47.45	33.63	0.03
Valeric acid	36.77	36.94	0.49
Hexanoic acid	9.89	9.51	0.42

### 3.8 Correlations between microbial communities, short-chain fatty acids, fatty acids, and average daily gain

Diets containing oat grain positively influenced growth performance, SCFAs, FAs metabolism, and the gut microbiota of lambs. We conducted a Spearman’s rank correlation analysis to investigate the microbiota potentially linked to SCFAs, FAs, and growth performance. At the phylum level (Figure 3E), *Bacteroidota* exhibited a negative correlation with valeric acid (*p* < 0.05).

*Acidobacteriota* and *Proteobacteria* were positively correlated with propionic, butyric, valeric, and hexanoic acids but negatively correlated with isobutyric and isovaleric acids (*p* < 0.05). *Acidobacteriota* also showed a negative correlation with C5:1 (*p* < 0.05). *Euryarchaeota* was positively associated with acetic, propionic, isobutyric, butyric, isovaleric, and hexanoic acids, and positively correlated with valeric acid (*p* < 0.05). *Gemmatimonadota* and *GAL15* were positively correlated with C9:0 (*p* < 0.05). *Spirochaetota* and *Synergistota* showed the strongest positive correlation with C5:1 (*p* < 0.01). *Acidobacteriota*, *Proteobacteria*, and *Chloroflexi* had the strongest positive correlation with ADG (*p* < 0.01), while *Actinobacteriota* was also positively correlated with ADG (*p* < 0.05). At the genus level (Figure 3F), *Bacteroidales_BS11_gut_group* was negatively correlated with propionic, valeric, and hexanoic acids (*p* < 0.05), whereas *Christensenellaceae_R.7_group* was positively correlated with hexanoic acid (*p* < 0.01). *Prevotellaceae_UCG.003* was negatively correlated with acetic, propionic, and hexanoic acids (*p* < 0.05) and had a highly significant negative correlation with valeric acid (*p* < 0.01). *Bacteroides* was negatively correlated with C9:0 (*p* < 0.05). Lastly, *Subgroup_2* was negatively correlated with C5:1 and C15:0 (*p* < 0.05) and positively correlated with ADG (*p* < 0.01).

## 4 Discussion

The intestine functions as the largest immune organ in an organism, serving as the primary site for nutrient digestion, absorption, and secretion (Vighi et al., 2008). The intestinal microbiota plays a pivotal role in maintaining homeostasis and overall health by modulating nutrient digestion and enhancing immune function (Lynch and Hsiao, 2019). The intestinal microbiota plays a crucial role in the host’s physiological, metabolic, and immunological processes, significantly influencing health and performance (Elmhadi et al., 2022). In ruminants, the ruminal bacterial community composition changes with dietary intake (Song et al., 2019). Investigating microbial communities and ruminal characteristics is essential for managing animal health and performance. The rumen microbiota is a complex ecosystem including bacteria, archaea, fungi, and protozoa, which convert plant materials into nutrients accessible to ruminants (Fu et al., 2022). These microbes facilitate the efficient utilization of starch and other non-fiber carbohydrates from grains and forages, allowing ruminants to extract more energy from fibrous feeds than monogastric animals (Zebeli and Metzler-Zebeli, 2012; Zhang R. et al., 2021). SCFAs, primarily acetate, propionate, and butyrate, are the main end products of feed digestion by rumen microorganisms. SCFAs serve as the principal energy source for ruminants and substrates for glucose and fat synthesis, supplying up to 70–80% of a ruminant’s total energy needs (Zhang J. et al., 2017). Moreover, fatty acids (FAs) are vital for various biochemical processes, with almost all FAs precursors in ruminant products produced in the rumen. As feed enters the rumen, dry matter is broken down into small molecules like SCFAs and peptides. Microorganisms utilize these substances to synthesize FAs, which are transported *via* the bloodstream and deposited in animal tissues. Thus, regulating the rumen environment can optimize FAs hydrogenation, increasing FAs content in products. The rumen microbiota is crucial in regulating nutrition and maintaining a stable rumen environment. Disruption of this microbiota balance can significantly impact nutrient digestion and utilization, affecting overall health and performance. The rumen microbiota’s adaptability to dietary changes makes it an ideal model for examining diet impacts on ruminant performance (Sun et al., 2022). Previous studies have highlighted the significant impact of diet on intestinal microbiota composition (Sun et al., 2022). Consequently, the gut microbiota is a vital link in the complex relationship between diet and the host’s health.

In this study, the body weight of the RO group of Hu sheep with added oat grain is significantly higher than that of the RC group, which warrants further investigation. According to the data in Table 1, the dietary energy of the two groups is almost identical, ruling out the possibility that uneven energy supply caused the weight difference. However, the water-soluble carbohydrate content in the RC group was higher than that in the RO group. Generally, water-soluble carbohydrate is an important energy source for animal growth, but our experimental results indicate that the RO group of Hu sheep did not experience growth inhibition due to the reduced water-soluble carbohydrate content, instead, their weight significantly increased. This phenomenon may be related to the higher fat content in the RO group. Fat, as another important energy source, may to some extent compensate for the deficiency in carbohydrate content. Additionally, as shown in Figure 1A, the feed intake of both groups of Hu sheep was almost the same, but the body weight of the RO group was significantly higher than that of the control group. This further indicates that the RO group with added oat grain exhibits higher efficiency in feed conversion. In summary, although the RO group has lower carbohydrate content, its higher fat content, and better feed conversion rate may be key factors contributing to its weight gain.

We employed 16S rRNA gene sequencing to analyze the intestinal microbiota of Hu lambs following dietary supplementation with oat grain. Consistent with prior research, the predominant phyla in the RO and RC groups were *Bacteroidota* and *Firmicutes*, respectively (Ye et al., 2016; Zhang R. et al., 2017). These phyla are crucial components of the rumen microbiota and are known for SCFAs production, such as acetate (Magne et al., 2020; Yue et al., 2021). Our Spearman correlation analysis revealed a negative association between *Bacteroidota* and acetic, propionic, and butyric acids. The observed decrease in *Bacteroidota* following oat grain supplementation contributed to an increase in SCFAs, which act as signaling molecules regulating lipid metabolism. An elevated *Firmicutes/Bacteroidota* ratio is associated with increased body weight. The inclusion of oat grain resulted in a significant reduction in microbial abundance, particularly Bacteroides, leading to an elevated *Firmicutes/Bacteroidota* ratio and increased final body weight in the RO group. This finding aligns with existing literature indicating that Firmicutes are linked to obesity, whereas *Bacteroidota* is associated with leanness (Ley et al., 2006).

Spearman’s analysis further confirmed that oat grain supplementation promotes weight gain in Hu lambs, as evidenced by the negative correlation of *Firmicutes* and *Bacteroidota* with ADG. Oat grain reduced the levels of both *Firmicutes* and *Bacteroidota* while simultaneously increasing lamb’s weight. *Firmicutes* are particularly important for protein degradation, starch digestion, and SCFAs synthesis, which are crucial for cellulose breakdown in ruminants (Shin et al., 2019). Conversely, *Bacteroidota* is involved in carbohydrate fermentation, nitrogen utilization, and bile acid biotransformation, thereby maintaining gut homeostasis (Backhed et al., 2004; Hooper et al., 2001).

Previous research has demonstrated a negative correlation between *Bacteroidota* abundance and both body fat and weight (Ley et al., 2005; Turnbaugh et al., 2008), consistent with our findings. Oat grain supplementation decreased *Bacteroidota* abundance while increasing body weight. Additionally, oat grain increased the abundance of *Acidobacteriota* and *Proteobacteria*, which were positively correlated with ADG and negatively correlated with isobutyric acid. *Acidobacteriota* also showed a significant negative correlation with citraconic acid, an indicator of undigested protein. The RO group exhibited lower isobutyric acid levels, suggesting improved protein digestion.

*Actinobacteriota* plays a crucial role in maintaining intestinal homeostasis and organic matter degradation, enhancing immune defense (Binda et al., 2018). Bifidobacteria, a member of *Actinobacteriota*, improve polysaccharide breakdown and hinder cholesterol absorption (Pereira and Gibson, 2002; Sonnenburg et al., 2006), positively correlating with body weight (Collado et al., 2008; Kalliomaki et al., 2008; Schwiertz et al., 2010). Oat grain supplementation increased *Actinobacteriota* abundance, consistent with the observed weight gain in Hu sheep. Spearman analysis revealed a positive correlation between *Actinobacteriota* and ADG, indicating its role in enhancing growth. *Actinobacteriota* can produce SCFAs, such as butyric acid and valeric acids, which enhance nutrient digestion and absorption.

Changes in the abundance of *Actinobacteriota* led to increased concentrations of SCFAs, such as butyric and valeric acids (Rodrigues et al., 2012). In our study, Spearman analysis revealed a positive correlation between *Actinobacteriota* and butyric, valeric, and hexanoic acids, while a negative correlation was observed with citraconic acid and pentadecanoic acid. Butyric acid is known to enhance levels of reactive oxygen species and mitochondria in muscle, thereby inhibiting muscle atrophy (Ley et al., 2006). The inclusion of oat grain in the diet promoted an increase in *Actinobacteriota* in Hu sheep, which subsequently enhanced body weight and SCFAs concentrations, such as valeric acid butyrate. These SCFAs facilitate nutrient digestion and absorption, maintain intestinal health, and improve overall productivity in animals.

The *Oscillospiraceae* family, capable of producing SCFAs, decreased significantly in the RO group (Supplementary Table S6). This family is prevalent in both human and animal intestines and is linked to obesity, weight loss, and gallstones, with a positive correlation to lean body mass index(Goodrich et al., 2014). In our study, oat grain supplementation in Hu lambs reduced *Oscillospiraceae* abundance, leading to decreased SCFAs, including acetic acid, and consequently, weight gain. There was an increase in *Prevotella* in the RO group (Supplementary Table S4). Conversely, *Prevotella* increased in the RO group. Known for its ability to break down cellulose and xylan, *Prevotella* ferments sugars to produce propionate, aiding starch, cellulose, and protein digestion. It synergizes with other bacteria to enhance nutrient degradation, improving fiber digestion and absorption.

To understand metabolic pathway differences in the intestinal microbiota of Hu sheep after oat grain dietary supplementation, we performed functional predictions and recorded microbial abundance. The results showed a significantly higher number of genes associated with lipid and amino acid metabolism for the RO group than the RC group. Lipid metabolism is crucial as it supplies animals with large amounts of energy, and facilitates energy acquisition and nutrient utilization. Meanwhile, amino acid metabolism enhances the production of SCFAs, such as acetic acid, which provides energy to the host (Neis et al., 2015). The metabolic pathways enriched in the intestinal tract of Hu sheep after incorporating oat grain are essential for growth and development. Therefore, the RO group has a greater capacity for nutrient absorption and utilization compared to the RC group.

Our study observed a higher content of SFAs in the RO group, though the difference was not statistically significant. This suggests that oat grain may influence the gastrointestinal microbiota in Hu sheep, leading to increased SFAs deposition in the rumen, which is then transported and deposited in tissues, potentially improving meat quality. Notably, azelaic acid, a type of SFAs, was significantly higher in the RO group. Azelaic acid is known to enhance intestinal permeability and reduce inflammation by activating the ectopic olfactory receptor 544 and stimulating GLP-1 secretion (Wu et al., 2021). The RO group also showed lower MUFAs levels, such as oleic acid, compared to the control group. This could be due to oat grain inhibiting the activity of butyivibrio and others partially involved in PUFAs hydrogenation, thereby reducing MUFAs content (Bhattacharjee et al., 2020). Adding probiotics to the diet may further regulate gut flora, inhibit hydrogenating bacteria, increase SFAs content, and reduce MUFAs deposition.

## 5 Conclusion

In summary, incorporating oat grain into Hu lamb’s diet significantly enhances their body weight and enriches microbiota diversity, particularly increasing the abundance of *Acidobacteriota*, *Proteobacteria*, *Chloroflexi*, and *Actinobacteriota*. It also reduces isobutyric acid and citraconic acid levels while increasing azelaic acid. Additionally, oat grain supplementation decreases the abundance of *Bacteroides* and *Oscillospiraceae*. These alterations are closely related to the growth performance of Hu sheep. Overall, incorporating oat grain into the diet affects microbiota composition, SCFAs, and FAs, ultimately impacting lamb’s growth.

## Data Availability

The original contributions presented in the study are included in the article/Supplementary material, further inquiries can be directed to the corresponding authors.

## Glossary

RC: Control group
RO: Oat-supplemented group
SCFAs: Short-chain fatty acids
FAs: Fatty acids
PUFAs: Polyunsaturated fatty acids
MUFAs: Monounsaturated fatty acids
DM: Dry matter
ADG: Average daily gain
ADFI: Average daily feed intake
CTAB: Hexadecyl trimethyl ammonium bromide
ASVs: Amplicon sequence variants
PCoA: Principal coordinate analysis
AA: Acetic acid
PA: Propionic acid
BA: Butyric acid
IBA: Isobutyric acid
VA: Valeric acid
IVA: Isovaleric acid
HA: Hexanoic acid
KEGG: Kyoto Encyclopedia of Genes and Genomes
C9:0: Azelaic acid
C18:1: Oleic acid
C8:0: Caprylic acid
C5:1: Citraconic acid
C15:0: Pentadecanoic acid
