# Supplementary material for: Impact of oat grain supplementation on growth performance, rumen microbiota, and fatty acid profiles in Hu sheep
Source: Front Microbiol. 2025 Feb 26;16:1528298. doi: 10.3389/fmicb.2025.1528298 (PMC11907649; doi:10.3389/fmicb.2025.1528298)
Supplement: Supplementary file 1 [file Data_Sheet_1.DOCX]

Supplementary Material

# Supplementary Data

**Table S1:** **Data processing statistical results**

| Sample Name | Raw PE | Combined | Qualified | Nochime | Base(nt) | AvgLen(nt) | GC% | Effective% | |
| --- | --- | --- | --- | --- | --- | --- | --- | --- | --- |
| RC1 | 77335.00 | 76175.00 | 76132.00 | 60530.00 | 15303994.00 | 253.00 | 55.02 | 78.27 |  |
| RC2 | 84047.00 | 81772.00 | 81724.00 | 66319.00 | 16768149.00 | 253.00 | 54.31 | 78.91 |  |
| RC3 | 73964.00 | 72011.00 | 71943.00 | 61642.00 | 15591025.00 | 253.00 | 54.62 | 83.34 |  |
| RC4 | 86586.00 | 84464.00 | 84375.00 | 67790.00 | 17145074.00 | 253.00 | 53.88 | 78.29 |  |
| RC5 | 79927.00 | 79376.00 | 79297.00 | 62650.00 | 15843131.00 | 253.00 | 54.19 | 78.38 |  |
| RC6 | 73710.00 | 73124.00 | 73069.00 | 61047.00 | 15440634.00 | 253.00 | 54.12 | 82.82 |  |
| RO1 | 84636.00 | 82450.00 | 82378.00 | 69307.00 | 17520609.00 | 253.00 | 54.28 | 81.89 |  |
| RO2 | 74461.00 | 73912.00 | 73871.00 | 60655.00 | 15332049.00 | 253.00 | 54.76 | 81.46 |  |
| RO3 | 74014.00 | 72654.00 | 72618.00 | 61056.00 | 15437273.00 | 253.00 | 54.11 | 82.49 |  |
| RO4 | 85331.00 | 84418.00 | 84358.00 | 66614.00 | 16843763.00 | 253.00 | 54.51 | 78.07 |  |
| RO5 | 82957.00 | 80739.00 | 80688.00 | 65025.00 | 16449399.00 | 253.00 | 54.52 | 78.38 |  |
| RO6 | 87551.00 | 86230.00 | 86155.00 | 73847.00 | 18686374.00 | 253.00 | 54.47 | 84.35 |  |

**Table S2: The alpha diversity index between the all samples**

| Sample Name | chao1 | dominance | goods_coverage | observed_otus | pielou_e | shannon | simpson |
| --- | --- | --- | --- | --- | --- | --- | --- |
| RC1 | 1234.261 | 0.008 | 1 | 1234 | 0.843 | 8.655 | 0.992 |
| RC2 | 1392.64 | 0.006 | 1 | 1389 | 0.85 | 8.878 | 0.994 |
| RC3 | 1170.652 | 0.006 | 1 | 1170 | 0.859 | 8.76 | 0.994 |
| RC4 | 1135.667 | 0.017 | 1 | 1132 | 0.791 | 8.021 | 0.983 |
| RC5 | 1242.476 | 0.007 | 1 | 1242 | 0.85 | 8.736 | 0.993 |
| RC6 | 1366.824 | 0.013 | 1 | 1366 | 0.814 | 8.477 | 0.987 |
| RO1 | 1504.053 | 0.006 | 1 | 1502 | 0.844 | 8.904 | 0.994 |
| RO2 | 1318 | 0.008 | 1 | 1313 | 0.834 | 8.64 | 0.992 |
| RO3 | 1346.677 | 0.006 | 1 | 1346 | 0.847 | 8.801 | 0.994 |
| RO4 | 1484.029 | 0.005 | 1 | 1483 | 0.854 | 8.996 | 0.995 |
| RO5 | 1546.621 | 0.008 | 1 | 1543 | 0.855 | 9.056 | 0.992 |
| RO6 | 1660.386 | 0.006 | 1 | 1658 | 0.854 | 9.133 | 0.994 |

**Table S3: The taxonomic distribution between RC group and RO group samples abundance at phylum level (top 10)**

| Taxonomy | RC1 | RC2 | RC3 | RC4 | RC5 | RC6 | RO1 | RO2 | RO3 | RO4 | RO5 | RO6 |
| --- | --- | --- | --- | --- | --- | --- | --- | --- | --- | --- | --- | --- |
| Bacteroidota | 0.600359 | 0.566305 | 0.554244 | 0.63556 | 0.468267 | 0.619319 | 0.621372 | 0.499972 | 0.387914 | 0.449751 | 0.467749 | 0.38747 |
| Firmicutes | 0.340097 | 0.348643 | 0.324115 | 0.318011 | 0.443702 | 0.267383 | 0.289303 | 0.339191 | 0.414421 | 0.365587 | 0.207173 | 0.410018 |
| Acidobacteriota | 0.001091 | 0.010414 | 0 | 0 | 0 | 0.01121 | 0.015889 | 0.04408 | 0.044561 | 0.05196 | 0.11931 | 0.048982 |
| Proteobacteria | 0.004014 | 0.008213 | 0.00653 | 0.00209 | 0.003089 | 0.024824 | 0.012874 | 0.028764 | 0.025212 | 0.031131 | 0.073417 | 0.049352 |
| Fibrobacterota | 0.00418 | 0.007362 | 0.042988 | 0.003478 | 0.004606 | 0.006474 | 0.00394 | 0.007103 | 0.012227 | 0.003552 | 0.005864 | 0.002719 |
| Spirochaetota | 0.017961 | 0.026174 | 0.029078 | 0.00862 | 0.039178 | 0.018794 | 0.013022 | 0.020477 | 0.024435 | 0.014539 | 0.014206 | 0.017832 |
| Chloroflexi | 0.000592 | 0.002442 | 0.000592 | 0.001646 | 0.001831 | 0.002571 | 0.003089 | 0.010192 | 0.011561 | 0.01034 | 0.023677 | 0.013466 |
| Patescibacteria | 0.004772 | 0.004217 | 0.004014 | 0.002423 | 0.008268 | 0.005494 | 0.002257 | 0.008046 | 0.023307 | 0.009378 | 0.003792 | 0.005142 |
| Actinobacteriota | 0.002053 | 0.003311 | 0.006585 | 0.006548 | 0.008823 | 0.004883 | 0.006585 | 0.010192 | 0.009582 | 0.010581 | 0.020958 | 0.014373 |
| Euryarchaeota | 0.013466 | 0.009859 | 0.005919 | 0.006807 | 0.00997 | 0.00727 | 0.006067 | 0.006974 | 0.016056 | 0.00812 | 0.007196 | 0.020902 |
| Others | 0.011413 | 0.013059 | 0.025934 | 0.014817 | 0.012264 | 0.031779 | 0.025601 | 0.025009 | 0.030725 | 0.04506 | 0.056658 | 0.029744 |

**Table S4: The taxonomic distribution between RC group and RO group samples abundance at genus level (top 10)**

| Taxonomy | RC1 | RC2 | RC3 | RC4 | RC5 | RC6 | RO1 | RO2 | RO3 | RO4 | RO5 | RO6 |
| --- | --- | --- | --- | --- | --- | --- | --- | --- | --- | --- | --- | --- |
| Prevotella | 0.141507 | 0.182091 | 0.134034 | 0.152957 | 0.147297 | 0.198443 | 0.245057 | 0.137733 | 0.127985 | 0.108359 | 0.233791 | 0.166553 |
| Rikenellaceae_RC9_gut_group | 0.107434 | 0.10773 | 0.093542 | 0.109765 | 0.091156 | 0.1869 | 0.042563 | 0.107878 | 0.089547 | 0.11537 | 0.067424 | 0.082277 |
| Bacteroidales_BS11_gut_group | 0.003607 | 0.008509 | 0.016666 | 0.154326 | 0.002701 | 0.018609 | 0.032796 | 0.012134 | 0.009101 | 0.006474 | 0.014724 | 0.003182 |
| F082 | 0.134607 | 0.076599 | 0.133886 | 0.07978 | 0.041509 | 0.082962 | 0.086069 | 0.064594 | 0.046854 | 0.069718 | 0.040861 | 0.028431 |
| Christensenellaceae_R-7_group | 0.043414 | 0.0357 | 0.036496 | 0.038531 | 0.065537 | 0.02475 | 0.031945 | 0.031132 | 0.026322 | 0.042323 | 0.021846 | 0.097057 |
| Prevotellaceae_UCG-003 | 0.012948 | 0.017129 | 0.01539 | 0.030632 | 0.003145 | 0.01441 | 0.08065 | 0.038586 | 0.021069 | 0.010618 | 0.013133 | 0.004069 |
| Muribaculaceae | 0.072326 | 0.05738 | 0.015593 | 0.015852 | 0.073565 | 0.013263 | 0.020384 | 0.057361 | 0.009711 | 0.043432 | 0.011321 | 0.036662 |
| Saccharofermentans | 0.034887 | 0.044616 | 0.026951 | 0.015205 | 0.056233 | 0.031058 | 0.018405 | 0.03657 | 0.03448 | 0.031668 | 0.023085 | 0.051238 |
| Subgroup_2 | 0.000407 | 0.003163 | 0 | 0 | 0 | 0.00431 | 0.005198 | 0.015557 | 0.015557 | 0.01872 | 0.043562 | 0.018701 |
| Fibrobacter | 0.00418 | 0.007362 | 0.042988 | 0.003478 | 0.004606 | 0.006474 | 0.00394 | 0.007103 | 0.012227 | 0.003552 | 0.005864 | 0.002719 |
| Others | 0.444683 | 0.459721 | 0.484453 | 0.399475 | 0.514252 | 0.418823 | 0.432992 | 0.491352 | 0.607147 | 0.549768 | 0.524389 | 0.50911 |

**Table S5: Effects of oat grain diet supplemen-tation on fatty acid contents in rumen of Hu sheep**

| Name | RO1 | RO2 | RO3 | RO4 | RO5 | RO6 | RC1 | RC2 | RC3 | RC4 | RC5 | RC6 |
| --- | --- | --- | --- | --- | --- | --- | --- | --- | --- | --- | --- | --- |
| Azelaic acid | 53.65382 | 68.49655 | 60.68746 | 48.02287 | 38.55816 | 56.26986 | 54.58567 | 36.03391 | 43.36103 | 33.97795 | 28.7095 | 36.95907 |
| Citraconic acid | 0.002571 | 0.005744 | 0.019776 | 0.001537 | 0.003443 | 0.009039 | 0.006066 | 0.009369 | 0.02234 | 0.009349 | 0.03842 | 0.017344 |
| Pentadecanoic acid | 3.04563 | 2.930745 | 2.261608 | 2.744669 | 2.196899 | 1.558817 | 4.38974 | 3.681477 | 3.844897 | 2.420605 | 2.8033 | 2.652283 |
| Tridecanoic acid | 0.420249 | 0.73811 | 0.680022 | 0.557396 | 0.298756 | 0.446116 | 0.859327 | 0.629336 | 0.948159 | 0.507405 | 0.722136 | 0.4523 |
| Myristic acid | 2.686128 | 3.359643 | 3.59671 | 3.170421 | 2.266664 | 1.678909 | 4.505617 | 3.128434 | 3.9855 | 2.981959 | 4.322757 | 2.241104 |
| Decanoic acid | 0.784013 | 0.558173 | 0.490793 | 0.355638 | 0.506235 | 0.990626 | 0.698546 | 0.720152 | 0.7713 | 0.47561 | 1.254742 | 0.841123 |
| Heptanoic acid | 12.19173 | 4.904216 | 3.930363 | 2.485739 | 4.314738 | 14.68276 | 12.11885 | 6.439087 | 8.827136 | 10.54542 | 11.20133 | 5.837625 |
| Dodecanoic acid | 0.724717 | 1.018966 | 1.760883 | 0.744923 | 1.041588 | 1.628676 | 1.564666 | 0.580318 | 1.711008 | 2.006492 | 3.085816 | 1.476798 |
| Itaconic acid | 0.442123 | 0.487376 | 0.228077 | 1.057479 | 0.169804 | 0.172228 | 0.24135 | 0.395034 | 0.227335 | 0.217589 | 0.167935 | 0.200404 |
| 2,2-Dimethyladipic acid | 0.47288 | 0.290546 | 0.510697 | 0.371872 | 0.227443 | 0.42808 | 0.444024 | 0.390942 | 0.381774 | 0.332051 | 0.515365 | 0.461253 |
| alpha-Linolenic acid | 1.402323 | 3.00703 | 3.015034 | 1.674456 | 0.720313 | 0.749759 | 2.194688 | 2.822122 | 3.146864 | 1.654219 | 1.487361 | 1.107642 |
| 10-Trans-Heptadecenoic acid | 0.040419 | 0.066917 | 0.420097 | 0.112741 | 0.042469 | 0.182343 | 0.374847 | 0.131981 | 0.074496 | 0.139089 | 0.152056 | 0.104672 |
| Octanoic acid | 9.275563 | 8.870125 | 7.69394 | 7.589451 | 10.18709 | 30.15766 | 12.11122 | 10.22004 | 12.79391 | 11.88806 | 18.779 | 12.6994 |
| Undecanoic acid | 0.126801 | 0.177336 | 0.18827 | 0.139642 | 0.117453 | 0.178225 | 0.184935 | 0.139112 | 0.191422 | 0.112257 | 0.225023 | 0.183107 |
| 12-Tridecenoic acid | 0.006438 | 0.011119 | 0.013132 | 0.005971 | 0.016414 | 0.002503 | 0.006021 | 0.000863 | 0.009287 | 0.0071 | 0.008402 | 0.011135 |
| Methylsuccinic acid | 3.947824 | 3.137426 | 5.313944 | 4.23043 | 3.499937 | 5.062978 | 2.927706 | 3.394166 | 3.421926 | 2.69877 | 5.878855 | 4.91375 |
| 3-Methyladipic acid | 3.827988 | 3.910363 | 5.437397 | 3.866649 | 1.703315 | 1.831497 | 4.018219 | 2.795849 | 3.262899 | 3.031266 | 1.691491 | 2.276557 |
| Nonanoic acid | 1.432594 | 1.472934 | 1.290753 | 1.300826 | 1.393585 | 2.164209 | 1.454709 | 1.23157 | 1.377399 | 1.574999 | 1.527535 | 1.30945 |
| Adipic acid | 9.10107 | 11.71493 | 10.41223 | 9.66419 | 4.915429 | 4.74161 | 9.27004 | 7.887344 | 8.603426 | 5.440451 | 5.984163 | 6.314955 |
| Oleic acid | 27.08727 | 23.40995 | 21.75714 | 9.457319 | 9.165019 | 1.684101 | 12.29336 | 27.97227 | 34.75411 | 9.808187 | 30.14416 | 3.364168 |
| Pimelic acid | 3.253111 | 3.585904 | 4.157777 | 3.010927 | 1.733009 | 2.079988 | 4.007465 | 2.41647 | 2.90208 | 2.73359 | 1.680464 | 2.454414 |
| 5Z-Dodecenoic acid | 0.055086 | 0.033047 | 0.100572 | 0.094901 | 0.067844 | 0.213917 | 0.053122 | 0.052366 | 0.093384 | 0.058685 | 0.237031 | 0.189714 |
| Citramalic acid | 0.168975 | 0.304308 | 0.312814 | 0.28021 | 0.19188 | 0.658889 | 0.322397 | 0.279442 | 0.245671 | 0.234884 | 0.620547 | 0.325108 |
| Sebacic acid | 10.187 | 11.55989 | 11.9343 | 8.949034 | 5.097798 | 5.605776 | 12.19626 | 6.872651 | 9.093621 | 8.399701 | 5.859138 | 6.438541 |
| 9E-tetradecenoic acid | 0.012566 | 0.050138 | 0.133611 | 0.035832 | 0.037066 | 0.062543 | 0.035002 | 0.052478 | 0.109001 | 0.064187 | 0.095673 | 0.0144 |
| 12-Hydroxystearic acid | 0.681537 | 1.776073 | 0.650425 | 0.747872 | 0.191656 | 0.414483 | 1.152147 | 0.704083 | 1.152096 | 0.657863 | 0.779711 | 0.188938 |
| 2-Butenoic acid | 1.674412 | 4.537376 | 3.368613 | 5.83403 | 3.3522 | 6.872358 | 2.371861 | 4.166728 | 2.979268 | 2.99029 | 6.400995 | 4.373886 |
| 4-Methylhexanoic acid | 0.904106 | 1.485958 | 1.134432 | 0.932349 | 1.402077 | 2.053339 | 1.055476 | 0.689843 | 0.785504 | 0.543345 | 6.603331 | 3.119617 |
| 2-Methylhexanoic acid | 0.012594 | 0.039242 | 0.04921 | 0.012807 | 0.01244 | 0.004299 | 0.026254 | 0.032485 | 0.009868 | 0.016348 | 0.004004 | 0.019224 |
| Suberic acid | 5.259074 | 7.065013 | 7.754575 | 5.508864 | 3.180533 | 4.045698 | 8.443257 | 4.049432 | 6.072512 | 5.144171 | 3.352999 | 4.81528 |
| Palmitoleic acid | 1.403923 | 1.506242 | 1.122839 | 0.528512 | 0.503297 | 0.523767 | 1.565008 | 0.813729 | 2.469844 | 0.809961 | 0.178149 | 0.544068 |
| Methylglutaric acid | 0.868022 | 1.039222 | 1.255727 | 0.91126 | 0.53791 | 0.676655 | 1.192198 | 0.889524 | 1.122759 | 0.914396 | 0.58455 | 0.645534 |
| 2,2-Dimethylsuccinic acid | 0.914978 | 1.131754 | 1.147863 | 0.929757 | 0.634352 | 0.661756 | 1.241533 | 0.956852 | 1.072721 | 0.826951 | 0.585111 | 0.703081 |
| 10Z-Nonadecenoic acid | 0.077786 | 0.102743 | 0.144126 | 0.015735 | 0.060347 | 0.082822 | 0.13021 | 0.176024 | 0.173544 | 0.068951 | 0.0182 | 0.016556 |
| 2-Hydroxycaproic acid | 2.904835 | 3.639596 | 3.435383 | 2.70118 | 2.805886 | 2.38066 | 2.846412 | 1.669621 | 3.266493 | 4.173662 | 2.949199 | 3.443722 |

**Table S6: The taxonomic distribution between RC group and RO group samples abundance at family level (top 10)**

| Taxonomy | RC1 | RC2 | RC3 | RC4 | RC5 | RC6 | RO1 | RO2 | RO3 | RO4 | RO5 | RO6 |
| --- | --- | --- | --- | --- | --- | --- | --- | --- | --- | --- | --- | --- |
| Prevotellaceae | 0.218272 | 0.251105 | 0.19454 | 0.215164 | 0.201273 | 0.259281 | 0.393814 | 0.220566 | 0.196667 | 0.187788 | 0.29513 | 0.206692 |
| Rikenellaceae | 0.117478 | 0.116239 | 0.100646 | 0.121104 | 0.099869 | 0.194817 | 0.045782 | 0.113705 | 0.092858 | 0.119458 | 0.069348 | 0.08618 |
| Bacteroidales_BS11_gut_group | 0.003607 | 0.008509 | 0.016666 | 0.154326 | 0.002701 | 0.018609 | 0.032796 | 0.012134 | 0.009101 | 0.006474 | 0.014724 | 0.003182 |
| F082 | 0.134607 | 0.076599 | 0.133886 | 0.07978 | 0.041509 | 0.082962 | 0.086069 | 0.064594 | 0.046854 | 0.069718 | 0.040861 | 0.028431 |
| Lachnospiraceae | 0.083757 | 0.091859 | 0.07388 | 0.061856 | 0.11537 | 0.062892 | 0.103513 | 0.112003 | 0.125266 | 0.079521 | 0.044542 | 0.102403 |
| Christensenellaceae | 0.044616 | 0.036237 | 0.036977 | 0.039381 | 0.066684 | 0.025231 | 0.032019 | 0.031409 | 0.026729 | 0.042748 | 0.02192 | 0.09813 |
| Muribaculaceae | 0.072326 | 0.05738 | 0.015593 | 0.015852 | 0.073565 | 0.013263 | 0.020384 | 0.057361 | 0.009711 | 0.043432 | 0.011321 | 0.036662 |
| Oscillospiraceae | 0.04617 | 0.043081 | 0.049537 | 0.065574 | 0.057509 | 0.041934 | 0.030188 | 0.032889 | 0.039381 | 0.036644 | 0.025268 | 0.046984 |
| Hungateiclostridiaceae | 0.036329 | 0.047798 | 0.029633 | 0.01613 | 0.058434 | 0.031594 | 0.019496 | 0.038401 | 0.03533 | 0.034813 | 0.023677 | 0.052552 |
| Ruminococcaceae | 0.037199 | 0.034332 | 0.026137 | 0.048149 | 0.038068 | 0.013984 | 0.02536 | 0.027617 | 0.0394 | 0.039104 | 0.009489 | 0.008805 |
| Others | 0.205638 | 0.236862 | 0.322506 | 0.182683 | 0.24502 | 0.255434 | 0.210577 | 0.289321 | 0.378702 | 0.340301 | 0.443721 | 0.329979 |
